# Smoking- and alcohol habits in relation to the clinical picture of women with microscopic colitis compared to controls

**DOI:** 10.1186/1472-6874-14-16

**Published:** 2014-01-23

**Authors:** Bodil Roth, Rita J Gustafsson, Bengt Jeppsson, Jonas Manjer, Bodil Ohlsson

**Affiliations:** 1 Department of Internal Medicine, Skåne University Hospital, Malmö, Lund University, Lund, Sweden; 2 Department of Gastroenterology, Skåne University Hospital, Malmö, Lund University, Lund, Sweden; 3 Department of Surgery, Skåne University Hospital, Malmö, Lund University, Lund, Sweden; 4 Department of Plastic Surgery, Skåne University Hospital, Malmö, Lund University, Lund, Sweden; 5 Department of Clinical Sciences, Skåne University Hospital, Malmö, Lund University, Lund, Sweden

**Keywords:** Alcohol habits, Collagenous colitis, Irritable bowel syndrome, Lifestyle factors, Lymphocytic colitis, Microscopic colitis, Smoking habits

## Abstract

**Background:**

Microscopic colitis (MC) induces gastrointestinal symptoms, which are partly overlapping with irritable bowel syndrome (IBS), predominately in middle-aged and elderly women. The etiology is unknown, but association with smoking has been found. The aim of this study was to examine whether the increased risk for smokers to develop MC is a true association, or rather the result of confounding factors. Therefore, patients suffering from MC and population-based controls from the same geographic area were studied regarding smoking- and alcohol habits, and other simultaneous, lifestyle factors, concerning the clinical expression of the disease.

**Methods:**

Women at the age of 73 years or younger, who had been treated for biopsy-verified MC at any of the Departments of Gastroenterology in Skåne, between 2002 and 2010, were invited to the study (240 patients). Women (737) from the population-based prospective cohort study, Malmö Diet and Cancer Study (MDCS), served as controls. A self-administered questionnaire about lifestyle factors, gastrointestinal symptoms, medical conditions and medication at the time for the study was sent by post.

**Results:**

Altogether, 131 women with MC could be included after age-matching with controls (median age 56 years) and exclusion of secondary MC. Patients were divided into persistent MC (MC1) and transient MC (MC2). Past smoking was associated with increased risk to develop MC2 (OR = 2.67, 95 CI = 1.15–6.23), whereas current smoking was associated with increased risk to develop MC1 (OR = 3.18, 95 CI = 1.57–6.42). Concomitant symptoms of IBS were associated with smoking (OR = 4.24, 95 CI = 1.92–9.32). Alcohol drinking had no association with MC or IBS.

**Conclusions:**

The results suggest that past smoking is associated with transient MC, whereas current smoking is associated with persistent MC. Smoking is associated with MC patients with concomitant IBS-like symptoms.

## Background

Microscopic colitis (MC) is an inflammatory disorder of the colonic mucosa, which causes gastrointestinal symptoms, but has a normal or near-normal endoscopic appearance [1]. Collagenous colitis (CC) (increased thickness of the subepithelial collagen band with or without increased intraepithelial lymphocytes (IEL)) and lymphocytic colitis (LC) (>20 IEL/100 enterocytes) are the two recognized forms [2]. Lymphocytic disorders of the gastrointestinal tract can be caused by a variety of different entities, e.g. infectious gastroenteritis, celiac disease, allergy, drugs, and surgical stretching of the tissue prior to biopsy sampling [3,4]. The MC diagnosis is set when the patient suffers from diarrhea in combination with histopathological characteristics [1,2]. The disease fluctuates with irregular relapses, and also the microscopic characteristics vary over time [2]. In a Swedish cohort, 63 had only a single attack of the disease [5]. Even so, histopathological findings of increased numbers of IEL often lead to the clinical diagnosis MC, and the patients who have once received the diagnosis MC are considered to suffer from a chronic disease [6]. Some studies have shown a female predominance in both CC and LC [1], mainly affecting middle-aged women, whereas others have not been able to confirm this in LC [5,7].

Previous studies have shown a correlation between past and current smoking and MC, in analogy with other subgroups of inflammatory bowel disease (IBD) [8-10]. We have recently shown that smoking impairs gastrointestinal- and psychological well-being in MC patients and is associated with IBS-like symptoms in addition to MC [11]. Reports indicate that MC is more frequent in northern countries. Epidemiological studies have shown a rising incidence in the past decade [1,10], in coincidence with the increasing wine consumption among women in northern countries during this time [12]. It is known that women are more vulnerable to alcohol than men [13].

The aim of this study was to examine whether the increased risk for smokers to develop MC is a true association, or rather the result of confounding factors. Thus, smoking- and alcohol habits, and other simultaneous, lifestyle factors, were compared between patients suffering from MC and population-based controls from the same geographic area, concerning the clinical expression of the disease.

## Methods

The study protocol for patients was approved by the Ethics Committee of Lund University, Dnr 2009/565 and 2011/209, and all participants gave their written, informed consent to take part in the study. The study protocol for controls was approved by the Ethics Committee of Lund University, Dnr 51–90.

### Patients

Women who had been treated for MC at any outpatient clinic of the Departments of Gastroenterology in the district of Skåne, between 2002 and 2010, were identified by search for the ICD-10 classification of the two forms CC and LC (K52.8) in outpatient records, as well as in the local register at the Department of Pathology, Skåne University Hospital, Malmö. About one-third of the total numbers of patients identified were excluded due to age over 73 years, as they had many other concomitant diseases and drug therapies. Of the patients recognized, only the 240 patients (median age 63 years, range 22–73 years) who had the diagnoses verified by colonic biopsy, and were under 73 years of age, were invited to participate in the present study. Altogether, 159 (median age 63 years, range 22–73 years) of the 240 patients invited accepted and were recruited to the study, but one patient was excluded due to another IBD diagnosis a few weeks after the inclusion, leaving 158 patients (66). Of these, 133 (55) also agreed to provide blood samples. These patients represent the majority of female cases of diagnosed MC in the southernmost districts of Sweden under the age of 73 years.

Both MC and IBS are more frequent in women than in men, and the quality of life and the perception of symptoms differ between the genders [1,14]. For this reason we chose to include only women in the study.

### Controls

#### The Malmö Diet and Cancer Study (MDCS)

The MDCS, a population-based, prospective cohort study, invited all women in Malmö born between 1923 and 1950. Recruitment was carried out between 1991 and 1996, and 41 of eligible subjects participated. In all, 17,035 women completed the baseline examination [15]. This MDCS baseline examination included a dietary assessment; a self-administered questionnaire about marital status, education, employment, smoking habits, wine consumption, physical activity, medical conditions, and medication; anthropometric measurements; and the collection of blood samples [16]. Menopausal status was defined using information on previous surgery and menstrual status. The classification of pre-, peri- and postmenopausal women has been described in detail elsewhere [17]. Women selected as controls in a previous study on breast cancer were used in the present study as controls. In all, 737subjects (median age 56 years, range 45–73 years) were available and the only exclusion criterion was that they should not have had a previous breast cancer at baseline [18].

### Patient recruitment and study design

Between March and June 2011, invitations, including information about the study and the same questionnaire as sent to controls about marital status, education, employment, smoking habits, wine consumption, physical activity, medical conditions, and medication was dispatched by post to all 240 women identified as having MC. In addition, also questionnaires about gastrointestinal symptoms, psychological well-being and Rome III criteria were sent by post to the women with MC. The data collection concerned the status for the patients at the time of follow-up and inclusion in the study, and not the status at the time of diagnosis. They were invited to visit the outpatient clinics of the Departments of Gastroenterology, Skåne University Hospital, Malmö or at the Central Hospital in Kristianstad, to provide blood samples. A reminding letter was sent a month after the invitation letter to those who had not answered. Questionnaires were completed 1–3 weeks before the blood samples were collected. Medical records were scrutinized, and age, gastrointestinal symptoms, examinations, and treatments were recorded. Their diagnosis, either CC or LC, was registered. Patients were divided into two groups based on their clinical presentation of their MC. One group included patients with at least two episodes of watery diarrhea; and/or their dependence on long-term treatment of corticosteroids to maintain remission; and/or two pathological intestinal mucosa biopsies (MC1, n = 78). These criteria are in line with those suggested for diagnosing IBD [19]. The other group included patients who had had only one episode of severe diarrhea (diarrhea that rendered examination by colonoscopy), or had had a normal biopsy after the initial pathological intestinal biopsy, in combination with a clinical remission (MC2, n = 53). Patients with concomitant celiac disease (9 patients) or an acute gastroenteritis shortly prior to the diagnostic colonoscopy (4 patients) were excluded as they had an obvious organic explanation for the intestinal inflammation, and were considered to be suffering from secondary MC [3,4]. The patients were also divided into two groups depending on whether concomitant IBS-like symptoms were present or not. Patients were compared to controls from the MDSC study.

### Questionnaires about gastrointestinal symptoms in patients

#### Rome III criteria

The patients completed a shortened version of the Rome III questionnaire, including only IBS symptoms [20]. This questionnaire has been translated and validated into the Swedish language (Magnus Simrén and Anna Rydén). Patients who fulfilled the criteria for Rome III were classified as also suffering from IBS, but as their diagnosis was MC, we have, in accordance with its presence in IBD, called it IBS-like symptoms [6].

#### Visual Analogue Scale for Irritable Bowel Syndrome (VAS-IBS)

The VAS-IBS is a short, psychometrical test developed to measure the treatment response and well-being during the previous two weeks in patients suffering from IBS [21]. The questionnaires include nine items about gastrointestinal symptoms and psychological well-being. The seven items, *abdominal pain, diarrhea, constipation, bloating and flatulence, vomiting and nausea, perception of psychological well-being,* and *the intestinal symptoms’ influence on daily life* are graded on a scale from 0 to100 mm, with 100 mm representing the fewest symptoms/health. The two questions *urgency and feeling of incomplete evacuation of bowel passage* are answered by “yes/no”.

### Statistical analyses

The data were analyzed using the statistical software package SPSS for Windows^©^ (Release 20.0; IBM, NY, USA). The patients were significantly older, with a wider age range than the controls. Therefore, the 12 patients younger and the two patients older than the controls were excluded, as were patients with celiac disease and gastroenteritis (13 patients), leaving 131 of the original 158 patients for the statistical calculations comparing patients with controls. Thus, both controls and patients were within the same age range 45–73 years. First, the distribution of continuous variables (age, disease duration, body mass index (BMI), days of wine drinking/month, and minutes of physical activity/week) was tested using an one-sample Kolmogorov-Smirnov test. All distributions differed significantly (p < 0.05) from a normal distribution, and therefore the factors studied were categorized and the values were given as median (interquartile range). There was no difference between CC and LC in any patient characteristics (data not shown), in accordance with a previous systematic review [22]. For this reason, all calculations were performed irrespective of the diagnosis, CC or LC. Differences between groups were calculated by the 2-tailed Mann–Whitney *U* -test. Correlations were performed by the Spearman rank correlation test. Fisher’s exact test was used for categorical variables. The Kruskal-Wallis test was used to calculate differences in VAS-IBS between subgroups of smoking- and alcohol habits, and was the only calculation including all 158 patients. A p-value < 0.05 was considered statistically significant.

Age was divided into 5-year intervals. The cohort was divided into quartiles of the number of days of drinking wine/month and the average number of exercising minutes per week during the year. Smoking was divided into three categories: subjects who had never smoked, subjects who had stopped smoking, and current smokers, including both regular and occasional smokers at the time of inclusion in the study. Subjects who denied intake of beer, wine, and liquor during the previous month before completion of the questionnaire were defined as having no alcohol intake. All subjects were then divided into four groups: subjects not using tobacco and alcohol, subjects only drinking alcohol, subjects only smoking, and subjects both smoking and drinking alcohol. Employment was divided into three categories: employed, retired or others, where others included housewives, students and unemployed. Education was divided into having a university education or not. There were missing values in days of drinking wine/month, smoking- and alcohol habits, physical activity, and level of education, which were labeled each as a specific category. The first category was used as reference. Factors intended for study (independent variables), namely, smoking habits and smoking- and alcohol habits, were initially examined using an unconditional logistic regression to calculate odds ratios with 95 confidence intervals (OR with 95 CI). Analyses of smoking habits were then performed adjusted for age at baseline, physical activity, days of drinking wine/month, level of education, and type of employment, as these characteristics had > 5 percentage difference between controls and patients, except in the case of MC2, where level of education did not differ between patients and controls. Smoking- and alcohol habits were adjusted for age, physical activity, level of education, and employment, except for MC2, where no adjustment for level of education was performed. Calculations were first performed on the whole patient group compared to controls, and then separately for patients with primary, persistent MC (MC1), transient MC (MC2), patients with IBS-like symptoms in addition to MC, and patients without IBS vs. controls (dependent variables).

## Results

### Patient characteristics

In total, 131 women (median age 63 (59–67) years) with MC were included in the statistical calculations, CC was diagnosed in 82 patients (62.6) and LC in 49 patients (37.4) (Table 1). The IBS criteria were fulfilled in 43 patients (52.4) with CC, and in 25 patients (51.0) with LC. The duration of the disease, at the time of inclusion in the study and data collection, was 7 [3-14] years. Measurements of hemoglobin (Hb) in blood and C-reactive protein (CRP) in plasma were in the majority of patients within reference values. Besides MC and IBS, 36 suffered from hypertension, 24 from rheumatoid arthritis, and 17 from asthma or bronchitis. The most common drug treatments at the time of inclusion in the study were corticosteroids (32.1), proton pump inhibitors (26.0), antidepressant drugs of the type selective serotonin reuptake inhibitors (21.4), angiotensin-converting enzyme inhibitors or angiotensin II receptor antagonists (18.3), statins (17.6), thyroid hormones (17.6), and beta-blockers (16.0). Patients with drug treatments at inclusion were older than patients without treatment, (64.92 (60.00–68.34) years and 62.07 (55.55–64.24) years, respectively, p = 0.012).

**Table 1:** Patient and control characteristics

	**Controls N = 737**	**MC1 N = 78**	**MC2 N = 53**	**IBS N = 68**	**non-IBS N = 63**
**Age at study (years)**	56.16 (50.47–62.36)	62.73 (58.60–67.30)	63.78 (59.98–66.67)	62.68 (58.46–66.96)	63.78 (59.64–67.18)
**Age groups (%)**					
45-49	17.1	5.1	3.8	5.9	3.2
50-54	22.3	9.0	3.8	5.9	7.9
55-59	22.3	14.1	13.2	16.2	11.1
60-64	19.5	28.2	37.7	32.4	31.7
65-69	11.7	25.6	26.4	22.1	30.2
70-74	7.2	17.9	15.1	17.6	15.9
**Smoking habits (%)**					
Never smoked	42.3	30.8	22.6	22.1	33.3
Stopped smoking	29.9	29.5	47.2	30.9	42.9
Current smokers	27.8	39.7	30.2	47.1	23.8
**Days of wine drinking/month (%) missing data**	6.8	3.8	7.5	2.9	7.9
0-2	49.5	46.2	37.7	41.2	44.4
3-4	15.2	10.3	15.1	16.2	7.9
5-7	12.3	7.7	9.4	8.8	7.9
>7	16.1	32.1	30.2	30.9	31.7
**Smoking- and alcohol habits (%) missing data**	0.3	3.8	3.8	2.9	4.8
No use	16.1	11.5	22.6	8.8	23.8
Drinking	55.8	47.4	45.3	42.6	50.8
Smoking	7.2	17.9	5.7	17.6	7.9
Smoking and drinking	20.6	19.2	22.6	27.9	12.7
**BMI (kg/m**^**2**^**) missing (%)**	24.84 (22.55–27.79) 0	24.40 (21.50–28.20) 42.3	26.07 (24.06–29.66) 47.2	26.10 (23.35–29.93) 39.7	24.40(21.56–26.94) 49.2
**Min of physical activity/week (%) missing**	2.2	25.6	30.2	25.0	30.2
0-223	25.0	35.9	30.2	36.8	30.2
224-385	24.0	16.7	18.9	13.2	22.2
386-585	24.6	10.3	11.3	11.8	9.5
>585	24.3	11.5	9.4	13.2	7.9
**Married women (%)**	61.9	57.7	58.5	57.4	58.7
**Level of education (%) missing**	0	2.6	1.9	2.9	1.6
≤12 years at school	76.1	62.8	75.5	67.6	68.3
>12 years at school	23.9	34.6	22.6	29.4	30.2
**Employment (%)**					
Employed	65.7	47.4	39.6	47.1	41.3
Retired	26.5	48.7	50.9	45.6	54.0
Others*	7.9	3.8	9.4	7.4	4.8

The values for smoking- and alcohol habits are based on the month prior to the completion of the study questionnaires. *BMI* = Body mass index, *IBS* = Irritable bowel syndrome, *MC* = Microscopic colitis. * = Includes housewives, students and unemployed. Values are given as median (interquartile range).

Of the patients, 91.7 were born in Sweden, compared with 90.2 of the controls. More patients than controls had a university degree (p = 0.001). As the patients were older than the controls, more patients were retired (p < 0.001) (Table 1). There was no difference between MC1 and MC2 regarding concomitant IBS-like symptoms (Table 2). The group of patients who only smoked, with no intake of alcohol, had the lowest values (most symptoms) on all VAS-IBS scales, but reached statistical significance only on the scales *bloating and flatulence* (p = 0.011) and *the gastrointestinal symptoms’ influence on daily life* (p = 0.012).

**Table 2:** Association between microscopic colitis (MC) and irritable bowel syndrome (IBS)-like symptoms

	**MC1**	**MC2**
	**N = 78**	**N = 53**
**IBS (n,%)**	39 (50.0)	29 (54.7)
**non-IBS (n,%)**	39 (50.0)	24 (45.3)

MC1 = persistent, primary microscopic colitis, MC2 = transient microscopic colitis. The prevalence of IBS and non-IBS is given as the number (percentage) of the MC population. Fisher’s exact test, p = 0.722.

### Smoking- and drinking habits in the total patient group

Of the patients, 27.5 had never smoked, in contrast to 42.3 of the controls. The prevalence of current smokers was 35.9 of the MC patients and 27.8 of the controls, whereas the prevalence of women who had stopped smoking was 36.6 of the patients compared with 29.9 of the controls. Of the 48 patients who had stopped smoking, 20 patients had done so during the observation period 2002–2010. In the MC patients, 16.0 used no tobacco or alcohol, 46.6 used alcohol, 13.0 were current smokers, and 20.6 used both tobacco and alcohol (3.8 missing values). The values for the controls are shown in Table 1. There was an association of increased risk for both former and current smokers to develop MC, calculated on the whole group (OR = 1.88, 95 CI = 1.04–3.39 and OR = 2.71, 95 CI = 1.50–4.91, respectively). When calculated with respect to both smoking- and alcohol habits, only the group of smoking without concomitant alcohol intake was associated with an increased risk to develop MC (OR = 2.80, 95 CI = 1.19–6.63). There was no association between smoking habits and the number of days of wine drinking/month in patients (Table 3).

**Table 3:** Association between days of wine drinking/month and smoking habits in patients with microscopic colitis

	**Smoking habits**		
	**Never smoked**	**Stopped smoking**	**Current smoker**
	**N = 36**	**N = 48**	**N = 47**
	**n (%)**	**n (%)**	**n (%)**
**Days of drinking wine/month**			
**Missing value**	1 (2.8)	3 (6.2)	3 (6.4)
**0-2 days**	18 (50.0)	14 (29.2)	24 (51.1)
**3-4 days**	4 (11.1)	8 (16.7)	4 (8.5)
**5-7 days**	4 (11.1)	5 (10.4)	2 (4.3)
**> 7 days**	9 (25.0)	18 (37.5)	14 (29.8)

The prevalence of different quartiles of days of drinking wine/month is given as the number (percentage) of smokers corresponding to the given heading. Fisher’s exact test, p = 0.430.

There were only few patients and controls drinking beer and stronger alcoholic beverages, albeit with many missing values. Therefore, only the parameter of “number of days of drinking wine/month” was used in the adjustments. The majority of patients drank 1–2 glasses a day when drinking. Those who drank beer and stronger alcoholic beverages were those who drank wine most days in the month (data not shown).

### Subgroup analyses for microscopic colitis groups

There was no statistically significant difference between MC1- and MC2 patients regarding smoking habits (p = 0.128), days of drinking wine/month (p = 0.711) or smoking- and alcohol habits (p = 0.160) (Table 1). Although fewer patients in the MC2 group had a university degree and more were unemployed compared to MC1 patients, these differences were not statistically significant (p = 0.277 and p = 0.344, respectively). In the MC1 group, current smoking was the only risk factor for developing MC, whereas in the MC2 group, past smoking was associated with an increased risk of developing the disease (Table 4).

**Table 4:** Associations between smoking- and alcohol habits and microscopic colitis

	**MC1**		**MC2**	
	**N = 78**		**N = 53**	
	**Crude OR 95% CI**	**OR 95% CI**	**Crude OR 95% CI**	**OR 95% CI**
**Smoking habits**				
Never smoked (reference)	1.00	1.00	1.00	1.00
Stopped smoking	1.36 (0.75–2.47)	1.26 (0.58–2.70)	2.96 (1.45–6.01)	2.67 (1.15–6.23)
Current smokers	1.97 (1.12–3.45)	3.18 (1.57–6.42)	2.03 (0.94–4.34)	2.14 (0.86–5.37)
**Smoking- and alcohol habits**				
Neither smoking nor drinking (reference)	1.00	1.00	1.00	1.00
Drinking but not smoking	1.19 (0.56–2.54)	1.25 (0.52–2.97)	0.58 (0.28–1.19)	1.00 (0.42–2.42)
Smoking but not drinking	3.49 (1.42–8.57)	5.86 (2.08–6.47)	0.56 (0.15–2.07)	0.65 (0.12–3.34)
Both smoking and drinking	1.30 (0.55–3.08)	2.50 (0.92–6.84)	0.78 (0.34–1.80)	1.67 (0.59–4.70)
Missing value	“-”	“-”	“-”	“-”

The values for smoking- and alcohol habits are based on the month prior to the completion of the study questionnaires. *OR* = Odds ratio, *CI* = confidence interval, MC1 = persistent, primary microscopic colitis, MC2 = transient microscopic colitis. Calculations of smoking habits in MC1 were adjusted for age, physical activity, days of drinking wine/month, level of education, and employment, whereas calculations in MC2 were adjusted for age, physical activity, days of drinking wine/month and employment. Calculations of smoking- and alcohol habits were adjusted for age, physical activity, level of education, and employment in MC1 and age, physical activity, and employment in MC2.

“-”, data not shown.

### Subgroup analyses in patients with and without IBS-like symptoms

There were more smokers in the IBS group than in the non-IBS group (p = 0.021). The number of days drinking wine/month did not differ between groups (p = 0.508), but the smoking- and alcohol habits differed, with more patients who neither smoked nor drank alcohol in the non-IBS group (p = 0.019) (Table 1). Patients with and without IBS-like symptoms had the same level of education (p = 1.000) and employment (p = 0.588) (Table 1). Smoking was associated with an increased risk to develop MC with IBS-like symptoms, whereas in the group of MC patients who were free of symptoms of IBS, this association was not seen (Table 5).

**Table 5:** Association between smoking- and alcohol habits in relation to irritable bowel syndrome (IBS) in microscopic colitis

	**IBS**		**non-IBS**	
	**N = 68**		**N = 63**	
	**Crude OR 95% CI**	**OR 95% CI**	**Crude OR 95% CI**	**OR 95% CI**
**Smoking habits**				
Never smoked (reference)	1.00	1.00	1.00	1.00
Stopped smoking	1.98 (1.00–3.94)	1.71 (0.73–4.02)	1.82 (1.00–3.31)	1.96 (0.94–4.12)
Current smokers	3.25 (1.72–6.15)	4.24 (1.92–9.32)	1.09 (0.55–2.16)	1.66 (0.73–3.76)
**Smoking-and alcohol habits**				
Neither smoking nor drinking (reference)	1.00	1.00	1.00	1.00
Drinking but not smoking	1.40 (0.57–3.45)	2.44 (0.81–7.36)	0.62 (0.32–1.18)	0.69 (0.32–1.48)
Smoking but not drinking	4.49 (1.60–12.60)	8.57 (2.39–30.75)	0.75 (0.26–2.17)	1.28 (0.40–4.12)
Both smoking and drinking	2.48 (0.96–6.40)	5.84 (1.77–19.29)	0.42 (0.17–1.02)	0.88 (0.32–2.42)
Missing value	“-”	“-”	“-”	“-”

The values for smoking- and alcohol habits are based on the month prior to the completion of the study questionnaires. *OR* = Odds ratio, *CI* = confidence interval. Calculations of smoking habits were adjusted for age, physical activity, drinking habits, level of education, and employment. Calculations of smoking- and alcohol habits were adjusted for age, physical activity, level of education, and employment.

“-”, data not shown.

## Discussion

The main finding in the present study of female MC patients was that smoking was associated with an increased risk to develop persistent MC and MC with concomitant IBS-like symptoms, independently of other lifestyle factors, whereas smoking was not associated with the development of solely MC, without IBS symptoms.

Both past and current smoking has been described as a risk factor for developing MC [8-10]. Almost half of the study patients who had stopped smoking had done so during the observation period, after the diagnosis of MC had been set. The association between past smoking and transient MC may thus depend on the fact that the patients regained their health when they stopped smoking.

Smoking has also been described as a risk factor for developing post-infectious functional gastrointestinal disorders (FGID) [23] and overlapping syndromes between reflux diseases and FGID [24]. Although extensive research to find the etiology of IBS and other functional bowel diseases has been conducted over recent decades, very few studies have investigated the effect of smoking on functional disorders. One previous study has described that smoking rendered symptoms of functional dyspepsia, but not IBS [25]. Another study has shown that both visceral and peripheral pain is increased by smoking [26]. In our present study, there was no difference in smoking habits in MC patients without IBS-like symptoms compared to controls. Furthermore, smoking was the only lifestyle or social factor associated with IBS-like symptoms in this MC cohort [11]. If smoking promotes the development of IBS or FGID, there will also be more patients examined by colonoscopy, and thus intestinal inflammation may be caught either as a transient or permanent finding, as these patients consume many of the drugs associated with MC [1,3,27]. Future research has to find out the mechanisms of how smoking affects the gastrointestinal tract, and whether smoking is mostly associated with IBS, or whether there is a true association with MC, without simultaneous IBS-like symptoms.

Chronic alcohol exposure is known to have a number of deleterious effects on the intestinal mucosa. Alcohol may trigger relapses and pronounced symptoms in patients with already developed IBD [28]. In animal experiments, alcohol leads to increased oxidative stress, hyperpermeability, neuropathy, and dysbiosis, which favor and sustain local inflammation [29-31]. Surprisingly, few reports have been published concerning alcohol intake in MC, but an increased alcohol intake in this entity compared to controls has recently been shown [8].

Red wine contains phenolic compounds, which have been shown to affect the composition of human gut microbiota [32]. There is growing evidence for involvement of gut microbiota in the development of IBD [33,34]. Bacterial endotoxins are important for induction of gut permeability, which is closely linked to induction and perpetuation of inflammation in IBD [34,35]. Phenolic compounds of red wine down-regulated serum concentrations of several cytokines and inflammatory markers, whereas others were increased [36]. Thus, the effect of ethanol in red wine may be counterbalanced by phenolic compounds, and the women in the present study drank mainly wine. The question as to whether wine consumption is harmful to the gastrointestinal tract, or whether there is a protective effect against smoking when combining smoking and alcohol, in analogy with the protective effect by alcohol in inducing rheumatoid arthritis, deserves further study [37].

Irritable bowel syndrome mostly affects young people, and colonoscopy is avoided in this entity. However, when older women consult a physician for the same symptoms, a colonoscopy is requested in order not to overlook a malignancy. When scrutinizing the medical records, we found that several of the patients had suffered from IBS for decades, but a colonoscopy was not ordered until the patient had reached higher age, due to the risk of malignancy. This may make the peak age for the MC incidence falsely high, and means that, on the basis of the same gastrointestinal symptoms, the younger patients are given the diagnosis IBS, while the elder patients are diagnosed as having MC. The MC patients are considered as IBS patients if not examined by a colonoscopy, which obscures the true incidence and prevalence figures [6,38].

Although MC is considered to be a subgroup of IBD, conventional anti-inflammatory drugs, with the exception of budesonide, are not as successful in the treatment of MC as in the treatment of classic IBD [10]. The results of the present study showed differences between subgroups of MC when divided according to a chronic, relapsing disease and a transient, single attack, in contrast to when divided into CC and LC due to histopathological findings, as has also been described previously [22]. It is now suggested that the diagnosis IBD should not be set until at least two attacks of the disease have occurred, as one attack of IBD is impossible to differentiate from other causes of diarrhea, e.g. gastroenteritis [19]. We would like to propound the same approach in MC, and suggest greater circumspection before the clinical diagnosis MC is set. First, all secondary forms of the disease have to be excluded [3], then, relapses must be observed, which requires that the patient must be followed up before the definitive diagnosis is set. The present, liberal attitude to the setting of the diagnosis MC, weakens the trustworthiness of research on etiological factors. If we use the pathologist’s diagnosis as a clinical diagnosis, without considering drugs and other secondary causes, or transient single attacks, the prevalence figures will be misleading, because they are too high.

One limitation of this study is that the women are elderly, with many concomitant diseases and drug treatments, thus generating several confounding factors [39]. The IBS-like symptoms may be due to side-effects of drugs rather than being true IBS. However, patients with MC and older than 73 years were excluded, which may be a bias in the information. However, if also these older women had been included, many patients with mild to moderate dementia could have been included, which could yield less validity in the completion of the questionnaires. Furthermore, still more concomitant diseases and drug treatments, and thus more confounders, would have been introduced. Future research must involve younger patients with only MC, in prospective studies. Another limitation is that gastrointestinal symptoms vary over time, and this study examined the symptoms only once, at varying time intervals after the diagnosis was set. The strength of the study is its cross-sectional character, with patients and controls from the same geographic area. However, as the controls were invited from the general population, some of these may also suffer from MC and IBS. The MDCS cohort was an external control group, probably reflecting a rather healthy group, with fewer smokers than in the general population. This can render an increased risk for all observations compared to the patient group. Nevertheless, we have previously published our findings that it was the smokers in this MC cohort who fulfilled the IBS criteria [11]. Furthermore, the patients and the controls were not enrolled during the same time period, which may influence the answers to the questions, especially concerning their drinking habits.

## Conclusions

In this study, we found that in MC patients under the age of 73 years, smoking is associated with MC, independently of alcohol consumption and other lifestyle factors. Future research has to examine how smoking increases the risk to develop MC, and also whether smoking increases in particular the risk to develop IBS and IBS-like symptoms, in comparison with MC.

## Abbreviations

CC: Collagenous colitis; CI: Confidence interval; FGID: Functional gastrointestinal disorders; IBD: Inflammatory bowel disease; IBS: Irritable bowel syndrome; LC: Lymphocytic colitis; MC: Microscopic colitis; OR: Odds ratio.

## Competing interests

The authors declare that they have no competing interests.

## Authors’ contributions

BR made substantial contributions to the conception and design, acquisition of data and was involved in drafting the manuscript. RJG was involved in drafting the manuscript and made substantial contributions to the acquisition of data. BJ was involved in revising the manuscript critically for important intellectual content. JM made substantial contributions to the design of the study, participated in the interpretation of the statistical analysis and was involved in revising the manuscript critically for important intellectual content. BO conceived of the study, and participated in its design and coordination and helped to draft the manuscript. All authors have read and approved the final manuscript.

## Pre-publication history

The pre-publication history for this paper can be accessed here:

http://www.biomedcentral.com/1472-6874/14/16/prepub
